# PrEP implementation research in Africa: what is new?

**DOI:** 10.7448/IAS.19.7.21101

**Published:** 2016-10-18

**Authors:** Frances M Cowan, Sinead Delany-Moretlwe, Eduard J Sanders, Nelly R Mugo, Fernand A Guedou, Michel Alary, Luc Behanzin, Owen Mugurungi, Linda-Gail Bekker

**Affiliations:** 1 Department of International Public Health, Liverpool School of Tropical Medicine, Liverpool, UK; 2 Centre for Sexual Health and HIV AIDS Research (CeSHHAR), Harare, Zimbabwe; 3 Wits Reproductive Health and HIV Institute, University of Witwatersrand, Johannesburg, South Africa; 4 Kenya Medical Research Institute, Nairobi, Kenya; 5 Nuffield Department of Medicine, University of Oxford, Oxford, UK; 6 Center for Clinical Research, Kenya Medical Research Institute, Nairobi, Kenya; 7 Department of Global Health, University of Washington, Seattle, WA, USA; 8 Partners in Health Research and Development, Thika, Kenya; 9 Dispensaire IST, Cotonou, Bénin; 10 Centre de Recherche du CHU de Québec, Université Laval, Québec, Canada; 11 Ministry of Health and Child Care, Harare, Zimbabwe; 12 The Desmond Tutu HIV Centre, University of Cape Town, Cape Town, South Africa

**Keywords:** empowerment, key populations, HIV/AIDS, pre-exposure prophylaxis, prevention, Africa, adherence, implementation

## Abstract

**Introduction:**

Of the two million new HIV infections in adults in 2014, 70% occurred in sub-Saharan Africa. Several African countries have already approved guidelines for pre-exposure prophylaxis (PrEP) for individuals at substantial risk of HIV as part of combination HIV prevention but key questions remain about how to identify and deliver PrEP to those at greatest need. Throughout the continent, individuals in sero-discordant relationships, and members of key populations (sex workers, men who have sex with men (MSM), transgender women and injection drug users) are likely to benefit from the availability of PrEP. In addition, adolescent girls and young women (AGYW) are at substantial risk in some parts of the continent. It has been estimated that at least three million individuals in Africa are likely to be eligible for PrEP according to WHO's criteria. Tens of demonstration projects are planned or underway across the continent among a range of countries, populations and delivery settings.

**Discussion:**

In each of the target populations, there are overarching issues related to (i) creating demand for PrEP, (ii) addressing supply-side issues and (iii) providing appropriate and tailored adherence support. Critical for creating demand for PrEP is the normalization of HIV prevention. Community-level interventions which engage opinion leaders as well as empowerment interventions for those at highest risk will be key. Critical to supply of PrEP is that services are accessible for all, including for stigmatized populations. Establishing accessible integrated services provides the opportunity to address other public health priorities including the unmet need for HIV testing, contraception and sexually transmitted infections treatment. National policies need to include minimum standards for training and quality assurance for PrEP implementation and to address supply chain issues. Adherence support needs to recognize that social and structural factors are likely to have an important influence. Combining interventions that build self-efficacy, empowerment and social cohesion, with evidence-based individualized adherence support for PrEP, are most likely to be effective.

**Conclusions:**

Efficacy of tenfovir-based PrEP is proven but many issues related to implementation remain unclear. Here, we have summarized some of the important implementation questions that need to be assessed as PrEP is rolled out across Africa.

## Introduction

Of the two million new HIV infections in adults in 2014, 70% occurred in sub-Saharan Africa (SSA). With the scale-up of antiretroviral treatment (ART), the number of new infections has stabilized [1]; however, to reach UNAIDS 90:90:90 target, it will be necessary to both scale up treatment and further intensify prevention efforts [2]. Mathematical models suggest that 25% of future HIV investments should go to effective combination HIV prevention [3]. In 2015, WHO recommended the use of tenofovir-based pre-exposure prophylaxis (PrEP) in individuals at substantial risk of HIV as part of combination prevention [4]. This recommendation was based on a systematic review of 18 studies across a range of populations and settings [5]. The review found that PrEP was effective in reducing HIV risk across gender, PrEP regimen, dosing and mode of acquisition, and that increased adherence was associated with a demonstrable increase in PrEP effectiveness. There was no evidence that PrEP use was associated with risk compensation, increased pregnancy-related outcomes or of hormonal contraception effectiveness [5]. Among trials with adherence ≥80%, PrEP reduced risk of infection by 70% (RR=0.30, 95% CI: 0.21–0.45, *p*=0.001) [5]. Several African countries have already licensed tenofovir plus emtricitabine for PrEP [6], while others have started the guideline development process, but key questions remain about how to identify and deliver PrEP to those at greatest need.

### Maximizing the impact and cost-effectiveness of PrEP

Modelling studies suggest that the impact and cost-effectiveness of PrEP will be greatest when used by populations at highest risk of infection, that is, those that have a HIV incidence of about three per 100 person-years or higher [7]. Preliminary work by UNAIDS which aims to chart sub-national HIV incidence across southern and eastern Africa in men and women suggests that incidence is ≥3% in at least one age/sex group in Kenya, Lesotho, Mozambique, South Africa, Swaziland and Uganda [8]. Throughout the continent, individuals in sero-discordant relationships and members of key populations (sex workers, men who have sex with men (MSM), transgender women and injection drug users) are likely to benefit from the availability of PrEP. UNAIDS 2016–20 has set a global target of putting 3 million people on PrEP annually, focused particularly on key populations and people at high risk in high prevalence settings [9].

While the case for making PrEP available across the continent is clear, it is important to recognize that not all those at substantial risk of HIV acquisition will opt to start it. To make an informed choice, people (and their healthcare providers) need to be able to accurately perceive their risk of HIV as well as their ability to mitigate it, have access to accurate information about the effectiveness, benefits and possible harms, and have the support and resources to be able to tailor its use to their requirements and to adhere to it. The availability of information about PrEP across Africa has not been formally mapped but is likely suboptimal, both among general and key populations and among healthcare providers. How best to generate demand for PrEP to those at highest risk of infection, without further stigmatizing them (or PrEP use) is not well understood.

### PrEP demonstration projects in SSA

Since completion of the efficacy trials, tens of PrEP demonstration projects are underway or planned across Africa (see Table 1), with projects enrolling female sex workers (FSW), MSM, adolescent girls and young women (AGYW), as well as the general population across a range of countries. In addition, studies are addressing the use of PrEP as bridge in sero-discordant couples before the infected partner becomes virologically suppressed on treatment and/or has acute HIV infection [10].

**Table 1:** Summary of ongoing and planned PrEP demonstration projects in Africa (as of July 2016)

Project name	Type of project	Sites	Target population	Proposed enrolment	Dates
*Ongoing projects*					
Partners demonstration project	Demonstration project PrEP as a bridge to ART	Kenya, Uganda	HIV sero-discordant couples	1013 HIV sero-discordant couples	Ongoing since August 2013; to complete follow-up: June 2016. Initial results presented at CROI 2015
Choice for Adolescents Methods for Prevention in South Africa (CHAMPS)	Demonstration project (Pluspills, combined with 2 other projects)	South Africa	Heterosexual male and female adolescents aged 15–19	150	Ongoing since July 2011; Pluspills is expected to be completed in October 2016; UChoose is expected to be completed in June 2016
Sibanye Health Project: Comprehensive HIV Prevention Package for MSM in Southern Africa Pilot Study	Demonstration project (pilot and integrated in a preventive package)	South Africa	MSM	200 MSM	Ongoing since February 2015; expected completion in May 2016
Sisters Antiretroviral Therapy Programme for Prevention of HIV – An Integrated Response (SAPPH-IRe)	Open label (combined with TasP)	Zimbabwe	FSW	1200 FSW eligible for PrEP (500 women enrolled as June 2016)	Ongoing since July 2014; expected completion in mid-2016
Gender-Specific Combination HIV Prevention for Youth in High Burden Settings (MP3-Youth)	Demonstration project (within a combination prevention package)	Kenya	Adolescent men and women aged 15–24	Only women on PrEP (enrolling 1215 total)	Ongoing since November 2014; expected completion in November 2016
Benin Demonstration Project with CHU de Québec (Canada)	Demonstration project (combined with TasP)	Benin	FSW	250 in PrEP; 100 in TasP	Ongoing since October 2014; expected completion in January 2017
Senegal Demonstration Project with Reseau Africain De Recherche Sur Le Sida, University of Washington and Westat	Demonstration project	Senegal	FSW	275	Ongoing since mid-2015; expected completion in late-2016
TAPS Demonstration Project (Wits RHI)	Demonstration project (combined with TasP)	South Africa	FSW (≥18 years)	400 in PrEP; 300 in TasP	Ongoing since April 2015. Expected completion April 2017
LVCT Health and SWOP Kenya (IPCP-Kenya)	Demonstration project (within a combination prevention package)	Kenya	FSW (≥18 years), MSM (≥18 years), young women at high HIV risk (15–29 years)	2100 participants	Ongoing; started December 2015. Expected completion December 2016
POWER	Demonstration project (within a package including microbicides)	South Africa, Kenya	Adolescent girls and young women aged 16–24; women aged 25–29	1500	Ongoing, started July 2015, PrEP delivery cohort to begin late 2016. Expected completion June 2020
Anova Health Institute's Health4Men initiative	Demonstration project	South Africa, Lesotho, Tanzania, Swaziland, Thailand, Uganda	MSM	300	Ongoing; started December 2015. Expected completion early-2016
CAPRISA 082	Demonstration project (observational cohort study)	South Africa	Adolescent girls and young women aged 18–24; women aged 25–30	Total enrolment of 2500, PrEP uptake expected to be 750	Ongoing, started March 2016. Expected completion April 2021
Nigerian National Agency for the Control of AIDS	Demonstration project (combined with TasP)	Nigeria	Heterosexual HIV sero-discordant couples	Enrolling 600 individuals on PrEP	Ongoing; started late 2015. Expected completion in late 2017
*Planned projects*					
EMPOWER Consortium Demonstration Project	Demonstration project (within a combination package including violence prevention)	South Africa, Tanzania	Adolescent girls and young women aged 16–24	To be determined	Planned; expected start mid-2016
Seasonal use of PrEP in Mozambique	Demonstration project on periodical use of PrEP (feasibility phase)	Mozambique	Women and men	To be determined	Feasibility study planning underway
DREAMS	Implementation initiative	Kenya, South Africa, Uganda, Zimbabwe	Young women aged 18–24	15,119; 3000; 1000; 1451	Demonstration projects starting mid-2016
Right to care (under DREAMS)	Demonstration project	South Africa	Adolescent girls and young women	To be determined	Planned
Tambua Mapema-PLUS	Pilot study	Kenya	Sero-discordant couples including patients with acute HIV infection, identified at care seeking	75	Planned, expected start Q4 2016
Médecins Sans Frontières (MSF) International – South Africa PrEP Project	Demonstration project	South Africa	Adolescent girls (<24 years old) and MSM	To be determined	Planned
HealthRight PrEP Project – Kenya	Demonstration project	Kenya	Male sex workers (MSW)	To be determined	Planned
HPTN 082	Demonstration project (observational cohort study)	South Africa, Zimbabwe	Young women aged 16–25 years	600	Planned; start date pending ethics submission, expected start in July 2016. Expected completion in August 2018
UNICEF PrEP Demonstration Program	Demonstration project	South Africa, Brazil, Thailand	Adolescents	Total target 15,071 (South Africa 10,000; Brazil 2671; Thailand, 2400)	Planned; expected start end-2016. Expected completion 2021
Champs PlusPills (DTHF, University of Washington)	Demonstration project	South Africa	Adolescent girls and young women aged 16–25	To be determined	Planned
Church of Scotland PrEP Project	Demonstration project	South Africa	Adolescent girls who are pregnant	To be determined	Planned
P3: Private Sector Provision of PrEP	Demonstration project	South Africa and Zimbabwe	Young women aged 20–34	Planned enrollment is 10,000 in each country	Planned; expected start end-2016
IMPAACT 2009	Observational study	Malawi, South Africa, Uganda, Zimbabwe	Adolescent girls and young women aged 16–24 who are pregnant	To be determined	Planned; expected start early-2017. Expected completion in 2019/2020
MTN 034/IPM 045	Phase IIa open label (randomizing participants to either dapivirine ring or oral PrEP)	South Africa, Uganda, Zimbabwe	Adolescent girls and young women aged 16–17	300	Planned; expected start early-2017
*Completed projects*					
iPrEx OLE	Open-label extension	South Africa, (Brazil, Peru, Ecuador, Thailand, USA)	MSM and transgender women	1250 (across all sites)	Completed. Open-label extension of the iPrEx trial (*Results show that PrEP provides a high degree of protection against HIV infection, even for individuals who miss some daily doses; high Interest in PrEP; longer-term evidence of safety and efficacy; and no sign of increased risk behaviour among PrEP users)*
Partners PrEP OLE	Open-label extension	Kenya, Uganda	HIV sero-discordant couples	1262 (assigned to TDF or FTC/TDF)	Completed. Open-label extension of the partners PrEP trial; started in July 2011 and was completed in December 2012. (*Results upon OLE completion in December 2012 showed both TDF and TDF/FTC highly efficacious*) 85% estimated efficacy of TDF and 93% of FTC/TDF**
CDC 494 (TDF2 follow-up)	Open-label extension	Botswana	Heterosexual men and women aged 18–39	1219 total (611 assigned to take daily TDF/FTC pill; 608 assigned to placebo)	Completed. Open-label extension of the trial among heterosexual men and women *(Results presented in July 2015 show strong adherence, high drug levels and no HIV infections and support efforts to expand PrEP availability in the context of generalized epidemics in resource-limited settings*)
HPTN 067 (ADAPT)	Phase II open-label (comparing 3 timings for drug taking)	South Africa (Thailand, USA)	Women (South Africa); MSM and transgender women (Thailand & USA)	622	Started in August 2011 and was completed in December 2014; final analyses are underway. (*Results from women in Cape Town show daily dosing fostered better adherence, better coverage of potential sexual exposure, and more sustained use of PrEP by South African women. Most study participants had higher coverage of sex events and better adherence when they were assigned to the daily dosing arm*)

Adapted from AVAC's “Ongoing and Planned PrEP Demonstration and Implementation Studies” table, *www.avac.org/prepdemo* and *www.prepwatch.org*.

#### PrEP provision in FSW

FSW are less engaged in HIV prevention and care services than women in the general population [11–13]. Sex work is criminalized in much of Africa leading to anxiety about confidentiality and contact with authorities [14]. FSW frequently face discrimination from health providers when they do access services [15]. Multipronged structural and community-led interventions are required to increase access and subsequent retention in services [16–20]. PrEP implementation projects targeting FSW have been conducted or are underway in Benin, Kenya [21], Senegal, South Africa and Zimbabwe [22] (Table 1). Of note, South Africa has recently become the first country in Africa to announce that it will make antiretroviral therapy (ART)-based prevention available for sex workers [23]. In Benin, a comprehensive prevention package is being implemented among FSW, with PrEP and ART being offered to 250 HIV-negative and 100 HIV-positive FSW, respectively. Women are seen quarterly (as is the case in most demonstration projects) and adherence is monitored using self-report and pill count, as well as tenofovir blood levels for those on PrEP, and HIV viral load (VL) monitoring for those on ART. Given concerns that PrEP use may lead to reductions in condom use, and increased risk of sexually transmitted infections and pregnancy, the project will determine whether the use of PrEP results in decreased use of condoms by measuring Y chromosome DNA and prostate-specific antigen in vaginal fluid [24,25].

In Zimbabwe, PrEP is being administered as part of the SAPPH-IRe trial, a cluster randomized trial of a community empowerment intervention combined with onsite access to ART and PrEP, and supported by the “Adherence Sisters Programme”, which includes an adherence buddy programme and reminder SMS combined with active follow-up of defaulters [26]. The primary analysis will compare the proportion of all sex workers with a VL over 1000 copies/ml living in intervention and comparison communities [27]. The population-level impact and cost-effectiveness will be modelled.

#### PrEP provision among MSM

Where there are data, HIV-1 incidence among MSM in Africa is high and higher than among FSW in some settings [28–30]. The pervasive homophobia that extends across Africa [31] coupled with the widespread criminalization of sex between men has resulted in MSM being very poorly engaged with prevention and care across the continent [32]. In some African countries, there are clinics that provide tailored services for MSM and where they exist, these are likely to be the optimal delivery channel. In other countries, where there is no specific service provision, more creative solutions will be required to engage MSM with services. There is limited evidence that PrEP may be acceptable to MSM in Africa. Qualitative assessments during a phase I PrEP trial of four-month duration among MSM and FSW in Nairobi and Coastal Kenya in 2009–2010 found that while side effects were experienced early in the study these diminished over time, and that characteristics of pills could improve comfort and use. Social impacts such as stigma, rumours and relationship difficulties due to being perceived as HIV positive were prevalent; interventions to address HIV and ART stigma will be important in this context [33]. Three demonstration projects are in progress among MSM in Kenya and South Africa (see Table 1) while one more is planned and another has been completed.

#### PrEP provision among adolescents and young people

There are 10 demonstration projects either planned, completed or underway among AGYW (see Table 1) in Kenya, South Africa, Uganda and Zimbabwe. Young women (15–24) in east and southern Africa are an important population for PrEP implementation. They represent three of the four million young people living with HIV in SSA [34]. Participants in recent HIV prevention trials, who were recruited specifically because they felt themselves at high risk of infection, had annual HIV incidence of 5–9% [35,36]. Evidence from treatment scale up suggests that adolescents find it more difficult to adhere to treatment than adults [37] and may require increased adherence support, tailored to their age group and lifestyle. One completed trial of PrEP in young women from Cape Town demonstrated that this population was able to adhere to daily dosing when supported to do so [38]. Of note, younger women in this study adhered more reliably to daily rather than intermittent or event-driven dosing. Several projects that are enrolling AGYW are either underway or are in the final stages of planning, with sites in South Africa, Kenya, Zimbabwe and Tanzania (see Table 1).

As with any medication prescribed or offered to those under age 18, there will be questions related to who provides consent. The only licensed drug currently available for PrEP use is Truvada, and PrEP is currently only a licensed indication for those aged 18 and over. There may be reluctance among providers to include PrEP for off-label usage. Drug safety is also an issue; there are concerns about bone safety with long-term use in younger populations. CHAMPS Pills Plus is an ongoing open-label study examining the safety, feasibility and acceptability of daily oral Truvada as PrEP in HIV-negative adolescents.

Although there is recognition that more intensive adherence support for young people will likely be required, this needs to be scalable. Approaches under investigation include individual adherence counselling, “adherence support clubs”, which either meet in person or virtually through “whatsapp” or both, use of treatment buddies, SMS and counselling on tenfovir drug levels (HPTN 082, Pluspills). The EMPOWER project in South Africa and Tanzania is also supporting young women to negotiate PrEP use within their intimate partnerships and within the broader social context. The DREAMS initiative is offering PrEP to young women as part of a comprehensive prevention package in Kenya, South Africa, Swaziland, Uganda and Zimbabwe with the intention of supporting prevention uptake and adherence using structural and behavioural interventions.

#### PrEP provision in sero-discordant couples

There are three PrEP demonstration projects either completed or underway among sero-discordant couples (see Table 1) in Kenya and South Africa. Estimates of new HIV infections occurring within stable, heterosexual partnerships in Africa range from 30 to 60% [39–44]. WHO has provided guidance on use of PrEP for sero-discordant couples since 2013 [45,46], but the extent to which this has been incorporated into national guidelines across Africa is not clear. Identifying sero-discordant couples is critical as it allows for a range of treatment and prevention interventions, including those to allow safe conception [47] and to prevent transmission in pregnancy, tailored to the couple's particular situation. While combining ART and PrEP for couples creates a package of mutual support and presents HIV care facilities as favourable service delivery venues, the efficiency and acceptability of delivery through HIV care facilities for HIV sero-discordant couples has not been evaluated. The Partners demonstration project has enrolled 1013 HIV sero-discordant couples in Kenya and Uganda since 2013, with near elimination of HIV infection (effectiveness of 96%) [10]. Plans are underway to recruit 1200 couples in a PrEP demonstration project in Nigeria (see Table 1).

#### PrEP provision following acute HIV-1 infection diagnosis

While PrEP may be initiated by the HIV-negative partner in an established HIV sero-discordant partnership, identification of discordancy for the vast majority of cases assumes detection of prevalent HIV. The HIV-negative partner is at greatest risk of HIV-1 acquisition when the infected partner has acute or early HIV infection [48,49]. Increasingly, there is interest in using risk and symptom score screening algorithms combined with point-of-care qualitative RNA tests [50] to identify patients with acute HIV infection (AHI) at care seeking [51]. The opportunity to identify sero-discordancy based on AHI patients presents a new dimension for PrEP initiation, as HIV-1 has been acquired from outside the partnership, and the HIV-negative partner is at greatest risk of HIV acquisition. A study among 1500 patients seeking care for symptoms and screened for prevalent HIV and AHI will start in Coastal Kenya in the second half of 2016, and PrEP uptake among sero-discordant couples identified in the context of care seeking will be assessed.

## Discussion

In each of the populations discussed above, there are overarching issues related to (i) creating demand for PrEP for both potential users and for providers, (ii) addressing supply-side issues and (iii) providing appropriate and tailored adherence support [52]. It is widely agreed that PrEP will need to be delivered as part of a comprehensive package of HIV prevention services. The numerous demonstration projects now underway will evaluate different strategies for reaching the target populations and different approaches to implementation across populations and settings. Results of these projects then need to inform locally developed and context-specific policy recommendations which in turn facilitate delivery and uptake PrEP.

### Demand creation

A critical first step in creating demand for PrEP is the normalization of HIV prevention, without this the stigma which undermines many HIV prevention interventions will persist. MSM, FSW and young women are important advocates for PrEP; empowering them with correct knowledge is likely to strengthen community trust, mitigate against rumours, increase acceptance, uptake and adherence. While empowerment of key populations is critical, community-level interventions that engage sex partners, opinion leaders and the general population about their role in HIV prevention are equally essential. If PrEP users come to be perceived by the community as “high risk” or “promiscuous”, this could greatly undermine uptake.

Of note, there is concern among the sex work community internationally that promoting PrEP may undermine condom and community empowerment programmes where they exist [53,54], and that rolling out PrEP to some could distract from ensuring provision of services for all. In addition, FSW worry that it could “re-medicalize” prevention, undermining community initiatives. In some settings, there is potential for PrEP administration to be coercive (particularly if long-acting injectable preparations become available) and stigmatizing. Implementation of PrEP in sex workers needs to be in full collaboration with sex workers themselves and be sensitive to the local concerns and realities that women face. Consideration of the rights of sex workers (and all those eligible for PrEP) is critical when formulating national implementation plans [16].

Risk perception among young women is often poor even among those at high risk of infection and was one of the greatest barriers to uptake in some, but not all, of the earlier PrEP trials [55]. Creating demand for PrEP among young women will necessitate addressing barriers to prevention uptake that young people face more generally, in addition to providing information about the effectiveness and availability of PrEP specifically. Raising community awareness using messages that resonate with young women to improve knowledge and establish norms around PrEP use and maximize its acceptability will be critical. Use of social media, video and community outreach both to create demand and inform risk assessment is planned or underway in many of the demonstration projects listed in Table 1.

### Supply side

Critical to supply of PrEP is that services are *accessible* for all, including stigmatized populations. For sero-discordant couples, it may be possible to make PrEP available through existing treatment facilities, but this will not be appropriate for the majority of potential users. For key populations, PrEP needs to be made available through specific programmes tailored to their requirements as well as through primary care. Establishing accessible integrated services for young women and their partners will be critical to scaling-up PrEP to this group and provides the opportunity to address other public health priorities for young people, including the unmet need for contraception.

Testing for HIV is the gateway to both treatment and biomedical prevention interventions, including for PrEP. Although rates of testing are increasing, there are still many people in Africa who are unaware of their status [56]. Scaling-up testing through a range of community-based approaches including self-testing, house-to-house and work-based approaches, with support for linkage to services is required. Training of health providers both to provide PrEP and transform judgmental and stigmatizing behaviours is important. In many parts of the world, misconceptions among health providers about the effectiveness and side effects of PrEP, as well as the likelihood it will induce drug resistance have slowed uptake [57]. National policies need to include minimum standards for training and quality assurance for PrEP implementation, as well as addressing supply chain issues which secure drug availability [58].

Making antiretroviral-based PrEP available through prevention services will require a shift in how they are organized. Antiretroviral drugs are currently procured, distributed and monitored through treatment programmes. Services providing PrEP using outreach may require adaptation of pharmacy or healthcare regulations. First-generation products will likely require a prescription from a healthcare provider, regular re-supply and monitoring and surveillance to detect ART resistance. As increasing evidence of safety emerges, barriers to access will likely reduce (as happened with hormonal contraception) [59]. There are several new products in the pipeline [60] and services need to be positioned so that they can introduce these as and when they are approved.

Cost is a significant consideration particularly where there is a culture of underinvestment in prevention [3]. For key populations and AGYW, risk is heterogeneous both between and within countries and finding ways to identify and support those at highest risk will be critical. The use of risk score tools may be appropriate but needs to be carefully evaluated [61,62]. Studies addressing the issues of willingness to pay are planned in some settings globally although the consequences of paying for PrEP when treatment is freely available need to be considered.

### Adherence support

PrEP adherence has generally been better in recent trials and open-labelled studies, when the efficacy of PrEP was already known likely changing the motivation for participation [63]. Adherence to PrEP does not need be lifelong and it is only required to cover periods of high risk [63,64]. For MSM, 100% adherence is likely not required for prevention of transmission, whereas pharmacokinetic studies among women suggest that adherence will need to be more consistent [65–67]. Some studies are exploring the use of tenofovir levels to support adherence. While the scalability of drug levels to monitor PrEP adherence is unlikely to be feasible using existing technology, point-of-care urine assays for tenofovir are in development. The cost of these assays will likely range from $10 to $20 per test, which may be potentially affordable for PrEP implementation programmes in some settings [68–70].

Adherence interventions need to be responsive to the social and structural factors that are likely to have an important influence. Combining interventions that build self-efficacy, empowerment and social cohesion, with evidence-based individualized adherence support for both PrEP and ART, are likely to be effective and mutually reinforcing [71–74]. Interventions that work across the cascade are likely to be more scalable and cost-effective than those that work on only one aspect [75].

FSWs are highly mobile. Supporting continuation of access to PrEP (or ART) despite this mobility is likely to be critical. Drawing on differentiated care models from the treatment world where those stable on treatment can collect drugs less frequently should be considered (possibly in combination with confirmation of adherence through drug levels) [76]. Alternatively, medication refill groups that facilitate less frequent collection of drug by alternating collection among group members may be an option [77].

### Research monitoring and evaluation

Implementation research is underway to determine how to take PrEP delivery to scale across Africa in a way that is sustainable, durable, non-stigmatizing, cost-effective and has the greatest impact. Data are required on the relative merits and disadvantages, including costs, of different demand creation, delivery and adherence support models, as well as real-world patterns of PrEP usage (see Table 2). Leakage of drug from treatment programmes, particularly in the event that PrEP is socially marketed in some settings will be important. Determining the population-level impact will also be important – several modelling projects are already underway to do this and will be strengthened by increasing the availability of programme data. Ongoing research to increase the choice of agents for PrEP needs to remain a priority [60].

**Table 2:** Implementation research questions for different target populations

	FSW	MSM	AGYW	SD couples	Following acute infection	All
Demand side	How to create demand without stigmatizing FSW?	How can demand be generated safely in countries where MSM illegal and highly stigmatized	How to improve risk perception among AGYW? How to assess PrEP readiness?	Is PrEP for sero-discordant couples required if countries move to Test and Start? If so for how long? Should PrEP be considered in addition to ART for index cases to support safe conception and during pregnancy?	Is it feasible to recruit HIV-negative partners of individuals with acute infection for PrEP safely and timeously?	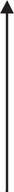
	How will availability of long-acting/injectable PrEP affect demand and uptake?					What combination of interventions most effectively (and cost effectively) support PrEP across the prevention cascade?
Supply side	What is the most appropriate venue for delivery of PrEP? What level of monitoring is required to safely deliver PrEP in the ‘real world’? What are the training and support requirements for healthcare workers delivering PrEP? What is the minimum training standard for all healthcare workers in use of PrEP? Are there specific supply chain issues in relation to provision of PrEP? What is the willingness to pay for PrEP in different settings/different populations? Does widespread use of PrEP affect population levels of ART resistance? Are there any other perverse effects associated with widespread implementation of PrEP (risk compensation/drug leakage from treatment programmes)?					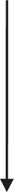
Adherence	Can community-based models of differentiated care to support use of ART be usefully tailored to support PrEP? What sort of adherence support is most effective for which groups? Including mHealth, adherence clubs, peer support? Does use of drug levels improve adherence counselling and support?					

## Conclusions

Efficacy of tenofovir-based PrEP is proven, but many issues related to implementation are still unclear. Here, we have summarized some of the important implementation questions that need to be assessed as PrEP is rolled out across Africa.
